# Beyond Institutionalization: Planning for Sustained Investments in Training, Supervision, and Support of Community Health Worker Programs in Bangladesh

**DOI:** 10.9745/GHSP-D-21-00156

**Published:** 2021-12-31

**Authors:** Shongkour Roy, Shivani Pandya, Md. Irfan Hossain, Timothy Abuya, Charlotte E. Warren, Paloma Mitra, Ubaidur Rob, Sharif Hossain, Smisha Agarwal

**Affiliations:** a Population Council, Dhaka, Bangladesh.; b Department of International Health, The Johns Hopkins Bloomberg School of Public Health, Baltimore, MD, USA.; c Population Council, Nairobi, Kenya.; d Population Council, Washington, DC, USA.; e Krieger School of Arts and Sciences, The Johns Hopkins University, Baltimore, MD, USA.

## Abstract

**Introduction:**

Bangladesh has a long history of mature and institutionalized community health worker (CHW) programs in primary health care. However, there is a lot of variability in the performance of the CHW programs in Bangladesh, as well as challenges associated with retention of CHWs. This study describes the challenges for CHWs, which in turn affect their motivation and performance.

**Methods:**

This study was conducted from December 2019 to January 2020 in 4 districts in Bangladesh: Cox's Bazar, Khulna, Rajshahi, and Sylhet. Twenty focus group discussions were conducted with 121 participants, including family welfare assistants (FWA), health assistants (HA), and their direct supervisors. Thirty in-depth interviews were conducted with upazila and district-level stakeholders. Data were analyzed using a thematic approach with a particular focus on CHW motivation, job satisfaction, and incentive preferences for improving morale and performance.

**Results:**

Several nonmonetary and monetary factors affect CHWs' motivation, performance, and job satisfaction. Recognition by the community, availability of promotions and technical recognition, increased training opportunities, reduced workloads, identification as government employees, access to transportation, provision of working tools, and improvements in the workplace environment were identified as important nonmonetary incentives. CHWs also discussed the importance of sufficient salaries and allowances.

**Discussion:**

Several factors impede the effectiveness of the CHW programs in Bangladesh. Changes to technical ranks and wages for the health care sector need to take a sector-wide lens to enable systematic calibration of wages for all health care workers. This study highlights that institutionalization of CHWs without adequate and sustained support for continued training, compensation, supervision, access to working tools, and recognition is insufficient to drive change. Identifying pragmatic strategies that can be supported through existing government budgets to address these factors is vital to sustaining the community health workforce in Bangladesh.

## Abstract (precis)

Institutionalizing community health workers (CHWs) is insufficient for improving program quality. Governments must plan for sustained investments for salaries and benefits, as well as systems enabling adaptive management of the CHW cadres. Greater coordination is needed at the global level to pool and align donor investments to support the ecosystem underlying CHW programs.

## Teaser

**Key Findings:**

- The community health worker (CHW) program in Bangladesh faces many systemic challenges related to inadequate technical recognition, training opportunities, identification as government employees, access to transportation, provision of working tools, and the workplace environment.
- The study highlighted tensions between family welfare assistants and health assistants owing to differences in their compensation structures despite comparable responsibilities—a situation that has resulted in demoralization of the cadres.
- Despite being institutionalized, the challenges faced by the CHW cadres in Bangladesh are very similar to CHW programs in other countries, highlighting the need to move beyond institutionalization to sustained investments in a supportive ecosystem for CHW programs.

**Key Implications:**

- Changes to technical ranks and wages for the health care sector need to take a sector-wide lens so that wages for all health care workers can be systematically calibrated.
- Efforts to institutionalize CHWs should be accompanied by plans for sustained investments in and support for training, adequate compensation, supervision, access to working tools, and recognition of CHWs.

## INTRODUCTION

Bangladesh has made great strides toward improving population health outcomes and meeting the Sustainable Development Goals, as evidenced by improvements in life expectancy at birth and under-5 mortality.^1^ Bangladesh is credited with creating one of the first community health worker (CHW) programs globally, shortly after the country gained independence in 1971.^2^ In 2019, there were over 185,000 CHWs. Approximately 70,000 were government based, with the remaining CHWs being supported by nongovernmental organizations and development partners (i.e., United Nations agencies).^3^^,^^4^ Such CHW programs play a critical role in facilitating access to care for the most marginalized populations and in addressing health inequities.

However, globally, as well as in Bangladesh, CHW programs continue to face challenges around high levels of attrition, irregularity in their activities, and poor overall performance.^5^ Systemic factors related to unwieldy workloads; insufficient supplies of drugs, vaccines, and other materials and equipment; lack of supervisory support; lack of promotions and upward mobility; and inadequate training all contribute to the challenges faced by these programs.^6^^–^^9^ An understanding of the incentives and the support received and valued by CHWs can help structure these programs to improve overall CHW motivation, satisfaction, and performance.

Understanding the incentives and support received and valued by CHWs can help structure programs to improve overall CHW motivation, satisfaction, and performance.

In Bangladesh, there are 3 paid government CHW cadres: family welfare assistants (FWA), health assistants (HA), and community health care practitioners (CHCP).^3^ FWAs are an entirely female workforce, whereas HAs and CHCPs include both men and women. FWAs provide counseling and promotion of family planning services under the Directorate General of Family Planning (DGFP); HAs support the Expanded Programme on Immunization (EPI), disease surveillance, and provide other primary health care services under the Directorate General of Health Services (DGHS); and CHCPs provide preventative and primary health care at the community clinics (CCs). CHCPs primarily manage the CCs, with FWAs and HAs spending 3 days out of their work week there; however, all 3 cadres' responsibilities overlap significantly in providing shared services to the community.^3^^,^^10^^,^^11^

Bangladesh's CHW program faces challenges with a reduced CHW workforce. One report noted that approximately “15% of government CHW positions are vacant at any given time.”^2^ The Government of Bangladesh's 2019 *National Strategy for Community Health Workers* report indicates that there are only 15,231 HAs, 19,583 FWAs, and 12,293 CHCPs, whereas the sanctioned posts for these positions are 21,000, 23,500, and 15,213 respectively.^3^ This shortage has led to both increased workloads for existing CHWs and larger catchment areas to cover, which has increased CHWs' overall dissatisfaction with their roles and responsibilities.^2^ Furthermore, “trends of feminization,” referring to when female CHWs leave their positions due to marriage or career changes, particularly affect CHWs within the family planning department (i.e., FWAs), given that it is an all-female cadre.^12^ These issues adversely affect not only CHWs but also the communities they serve and support; therefore, it is important to identify appropriate strategies to address these challenges to strengthen Bangladesh's community health program.

The *WHO Guideline on Health Policy and System Support to Optimize Community Health Worker Programs* identified that both nonmonetary and monetary incentives are important in supporting and motivating CHWs and can reduce CHW attrition.^5^ In their analysis of how incentives affect motivation for both salaried and volunteer CHWs across 6 countries (including Bangladesh), Ormel et al.^8^ provided further support for the value of both nonmonetary and monetary incentives, indicating that solely providing financial incentives is likely to be insufficient in maintaining motivation.^8^ Studies among nongovernmental organization CHWs in Bangladesh similarly identified the importance of providing monetary incentives, as well as social prestige, recognition, and community acceptance, among others, in improving CHW retention.^6^^,^^7^^,^^13^ A systematic review of 14 studies of interventions for CHW performance improvement found moderate-quality evidence toward the impact these interventions had on community health outcomes, noting improvements in specific behavioral outcomes, use of services, and quality of care provided.^14^ These findings emphasize the importance of providing both nonmonetary and monetary incentives as strategies toward improving CHW programs, which includes boosting CHW motivation, satisfaction, and performance.

In alignment with recommendations of prior research, Bangladesh's recent *National Strategy for Community Health Workers* report outlined the provision of monetary and nonmonetary incentives to better recognize and support CHWs.^3^ This represents a significant and crucial first step toward better working conditions for CHWs and aligns with the recent Astana Declaration, which underscored the importance of CHWs in advancing universal health coverage.^15^ Given the immense value of CHWs and the role they play in promoting and improving health outcomes in Bangladesh, it is necessary to identify ways to better motivate them and improve their working conditions through the provision of realistic and desirable incentives at the policy level. This work is especially critical now as the national government is currently in the process of deploying new CHW cadres and restructuring incentives for the existing cadres. This study provides a stronger understanding of the current CHW context in Bangladesh and their preferences for incentives, as well as the disincentives that increase dissatisfaction with their role.

Given the immense value of CHWs in promoting and improving health outcomes in Bangladesh, it is necessary to identify ways to improve their working conditions.

## METHODS

### Ethical Approval

The research protocol was approved by the Population Council's Institutional Review Board (PC IRB 872) and the Bangladesh Medical Research Council Institutional Review Board (BMRC/NREC/2016-2019/114).

### Study Setting and Population

**FIGURE 1:**
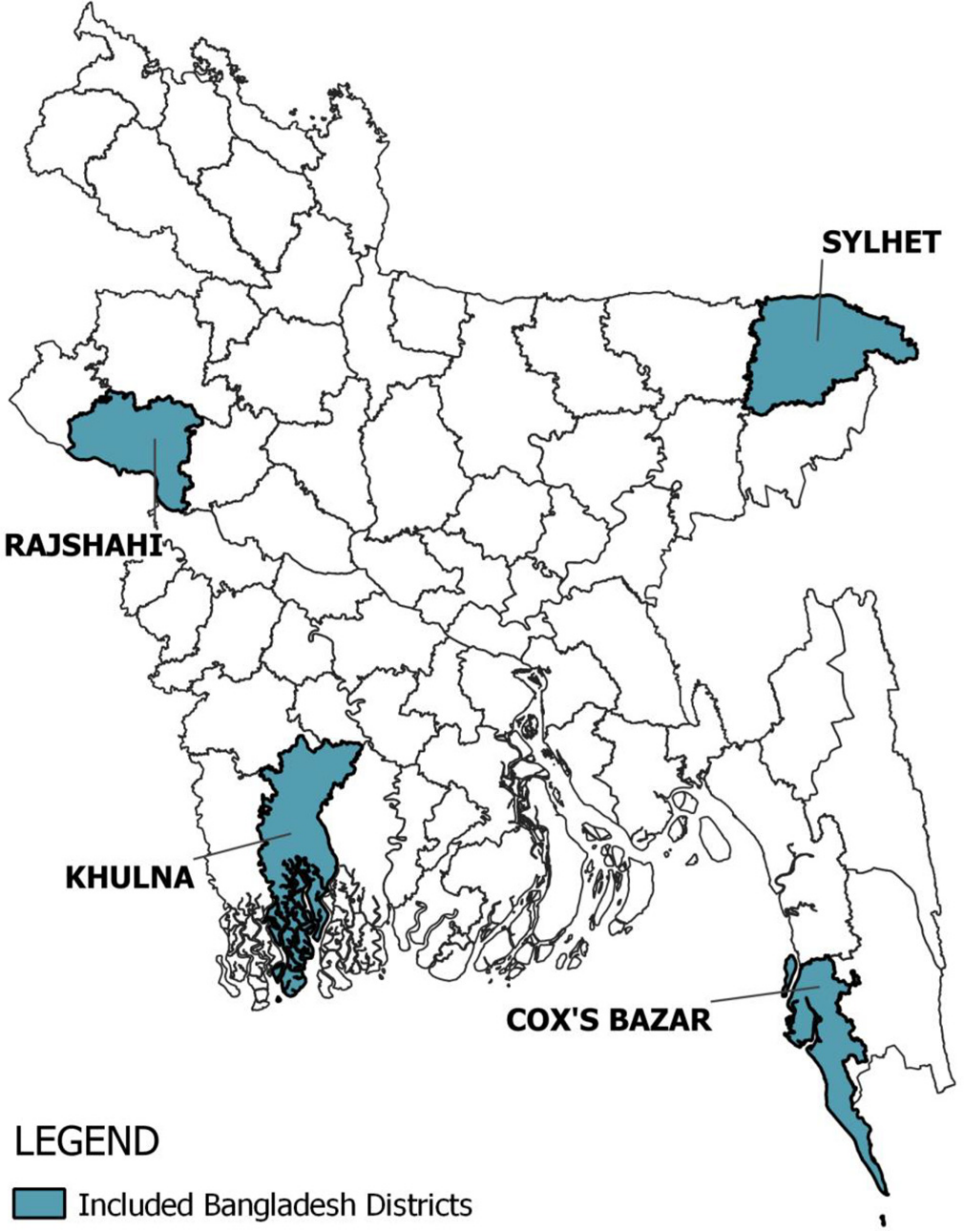
Map of Study Area of Community Health Worker Challenges Affecting Motivation Conducted in 4 Districts in Bangladesh: Cox's Bazar, Khulna, Rajshahi, and Sylhet

This study was conducted in 4 districts of Bangladesh: Cox's Bazar, Khulna, Rajshahi, and Sylhet. These study sites were selected to represent perspectives from different regions of Bangladesh (Figure 1).^16^ Cox's Bazar was selected as a study district given the high percentage of CHWs supporting the Rohingya refugee population in the district; over 720,000 Rohingya refugees migrated to Bangladesh in August 2017, which created the fastest-growing refugee crisis globally.^17^ Over 1,000,000 displaced Rohingya refugees now reside in camps located within 2 upazilas (administrative subunits) within the district: Ukhiya and Teknaf.^17^^,^^18^

**FIGURE 2:**
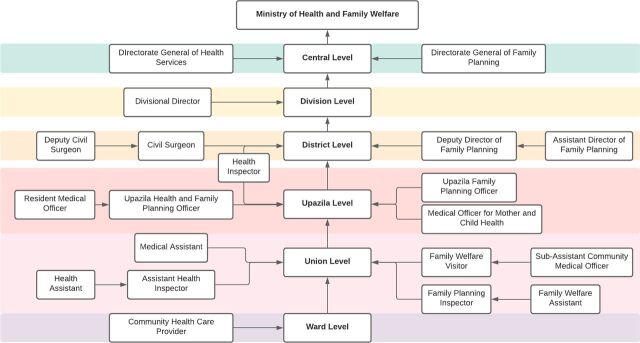
Bangladesh's Community Health System Structure

**FIGURE 3:**
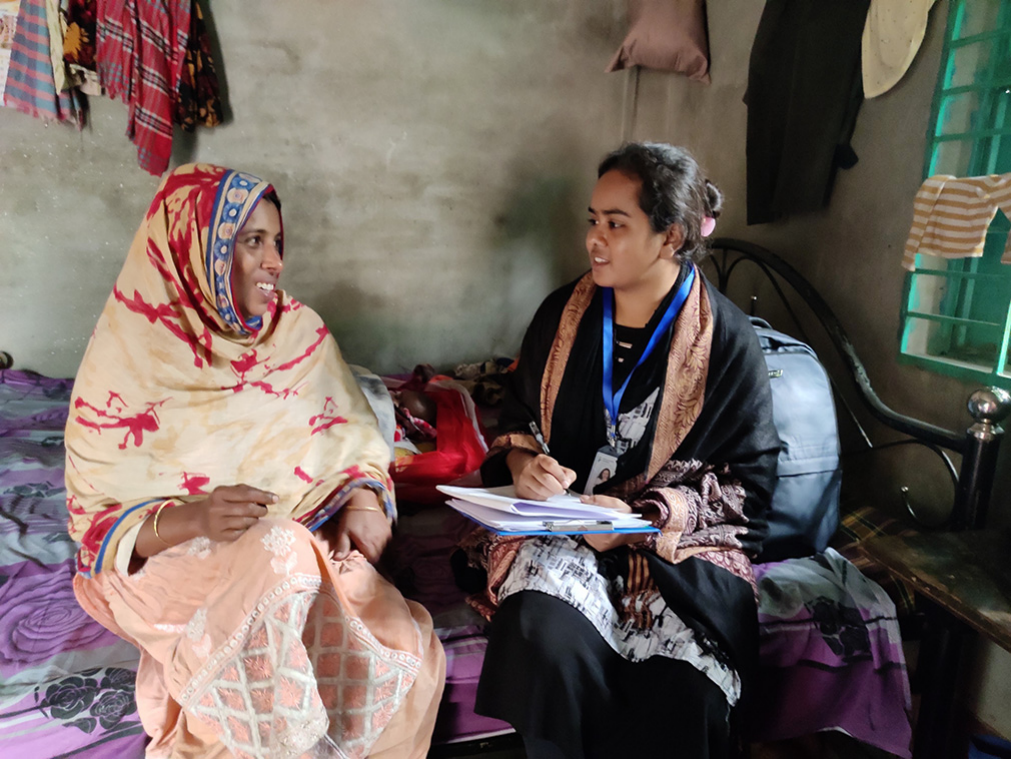
Bangladesh's Health Services and Family Planning Organizational Structure, With the Administrative Level

Participants were grouped into 3 categories: CHWs, CHW supervisors, and upazila and district-level stakeholders. CHWs included HAs and FWAs. CHCPs were not included in the study due to their role in primarily supporting the CCs; this study focused on CHWs working within the community and making home visits. CHW supervisors included assistant health inspectors (AHIs), health inspectors (HIs), and family planning inspectors (FPIs). AHIs and HIs operate as the direct supervisors for HAs under DGHS; AHIs are union level and HIs are upazila/district level.^3^ FPIs are the direct supervisors for FWAs under DGFP and serve at the union level.^3^ Upazila- and district-level stakeholders included civil surgeons, deputy directors of family planning, upazila health and family planning officers, and medical officers in maternal and child health, among others. Figures 2 and 3 provide an overview of Bangladesh's community health system structure.

**Figure:**
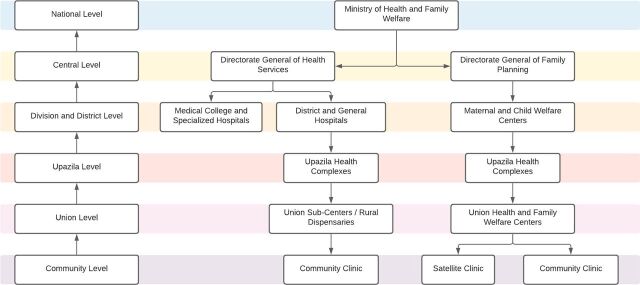
A community health worker in Bangladesh discusses incentive experiences, motivation, and job satisfaction with an interviewer. © 2020 Bobita Khatun/Population Council Bangladesh

In Bangladesh, CHWs and their supervisors are government employees, who receive government salaries and allowances. Currently, their salary pay scale is dictated by their grade position. Before July 2015, HAs and FWAs had a similar pay scale and both were in the third-class position. In July 2015, the Bangladesh government, through the eighth national pay scale, changed the class positions and grades for government employees; FWAs became 17th grade and HAs 16th grade. FWAs were effectively demoted, as their grade was one below HAs, which resulted in a change and decrease with the pay scale.

### Study Design and Data Collection

Focus group discussions (FGDs) were conducted with CHWs and CHW supervisors and in-depth interviews (IDIs) were conducted among upazila- and district-level stakeholders between December 2019 and January 2020. Twenty FGDs were conducted among 121 participants, which included HAs, FWAs, and their direct supervisors (HI, AHI, FPI), and 30 IDIs were conducted with upazila- and district-level stakeholders (i.e., civil surgeon, deputy directors of family planning, upazila health and family planning officers, upazila family planning officer); there were a total of 151 participants overall (the Table shows participant breakdown by district). Semistructured FGD and IDI guides were created to help guide the discussions. Questions focused on the types of incentives and disincentives that supported CHW motivation, satisfaction, and retention; the relationship between CHWs and health care facilities; the relationship between CHWs and the community; and government financial capacity and goodwill toward CHW programs.

**TABLE.** Number of Focus Group Discussions and In-Depth Interviews in a Study of CHW Challenges Affecting Motivation in Bangladesh, by District

District	No. of CHW FGD Participants	No. CHW Supervisor FGD Participants	No. of Policy-Level Stakeholder IDI Participants	Total
Cox's Bazar	12	12	7	31
Khulna	26	23	5	54
Rajshahi	12	12	9	33
Sylhet	12	12	9	33
**Total**	62	59	30	151

Abbreviations: CHW, community health worker; FGD, focus group discussion, IDI, in-depth interview.

The study team worked with DGHS and DGFP, under the Ministry of Health and Family Welfare, to initially orient them on the study protocol and to gain approval and access to health care facility staff. Participants were purposively selected. All participants provided informed consent before their participation. FGDs and IDIs were facilitated by trained postgraduate facilitators. FGDs had 2 facilitators, with one moderating the discussion and the second taking notes. The duration of the FGDs and IDIs ranged from 1 to 2 hours. The FGDs and IDIs were held in Bangla, audio-recorded, and subsequently transcribed and translated into English for analysis.

### Analysis

Data from the FGD and IDI transcripts were coded and analyzed using QSR International's NVivo 12 software.^19^ A thematic content analysis technique was applied, which comprised a mix of inductive and deductive coding.^20^ The coding framework was adapted from one initially developed for one of the 4 countries included in the larger study; through an iterative process, a team of 3 researchers read through the transcripts and identified emerging themes to include in the coding framework. To ensure inter-rater reliability, the researchers familiarized themselves with the data and the coding framework by coding 2 transcripts independently and discussing each together to come to a consensus on coding strategies. A constant comparison approach was applied to systematically compare and analyze the coded data to provide a comprehensive picture of incentives and to summarize the similarities and differences in the perception and experiences of incentives between different stakeholders and study sites.

## RESULTS

The discussions with the CHWs, CHW supervisors, and upazila/district-level stakeholders provided critical insights into factors that influence and inhibit CHW motivation, job satisfaction, and performance. The results are grouped into 2 categories: nonmonetary and monetary factors.

The FGDs and IDIs provided critical insights into factors that influence and inhibit CHW motivation, job satisfaction, and performance.

### Nonmonetary Factors

*Recognition by the Community, Health Care Facilities, and the Government*

Recognition of CHWs' responsibilities can help further motivate their job satisfaction and performance. CHWs reported generally positive relationships with and recognition by the community, with CHWs sometimes being referred to as doctors:

*They call us as doctors. If they have any problems, they come to us.* —CHW Supervisor FGD

CHWs' relationship with health care facilities was more strained, due to tensions between CHW cadres—specifically between FWAs and HAs/CHCPs. As aforementioned, FWAs are under DGFP, and HAs/CHCPs are under DGHS. Discussions suggested poor collaboration and lack of mutual support between FWAs and HAs/CHCPs that ultimately served to undermine FWAs ability to work effectively:

*There are CHCPs and HAs who work in community clinic. When FWAs are trying to collaborate with them, sometimes they are not getting proper recognition. It is a big issue to work [with] each other*. —Upazila/District-Level Supervisor IDI

Furthermore, CHWs noted that tangible forms of recognition need to be provided to them from the policy level, namely through promotions and technical recognition. Technical recognition refers to being provided technical training (e.g., to become a community skilled birth attendant), which can, in turn, improve their salary scale and credibility. Currently, regarding promotion, there is no scope for FWAs and limited scope for HAs, which has adversely affected their motivation.

*Yes, promotion. I joined in 1990. Thirty years have passed. This work feels boring now. I feel that if there was promotion for us, we would be happy with our work. I have worked all my life being in the same rank. Nowhere in Bangladesh you can see this situation, only in the family planning department.* —FWA/CHW FGD

*One employee passes 30–35 years working for the same post. Very few get the promotion based on the seniority level. […] They join the department as health assistant, also retire from the department as the health assistant. They do not get the opportunity to become AHI or HI. How cruel is this!* —CHW Supervisor FGD

### Identification

CHWs noted that methods of identification can help support their ownership of their work, as well as recognition by the community and health care facilities. CHWs discussed identification cards, branded uniforms (e.g., t-shirts, aprons), and branded working tools (e.g., umbrellas, carrier bags). CHWs shared that the lack of adequate identification resulted in difficulties in executing their responsibilities. Wearing something that identified them as a CHW would indicate their function in the communities and better allow them to perform their jobs more effectively.

*In Sadar, the FWAs don't have a uniform. It's like their identity. It represents them as FWA. Whenever they roam without their uniform, people get scared of them. It's a city, it's full of danger. People of Sadar often assume that they are frauds and do not want to believe them. The FWAs have to assure them saying that they are FWAs, they are from the government. How can they do their job without a basic apron?* —CHW Supervisor FGD

### Means of Transportation

CHWs often travel by foot in communities or must wait for transportation, which can increase the amount of time it takes to get around and complete their work. CHWs discussed the need to have bicycles or scooters/motorcycles, as it would help them be more mobile and increase their access to and time in communities, especially in remote, hard-to-reach areas. Additionally, it would help ease the weight they have to carry, given that CHWs carry their supplies with them.

*One has said there should be one HA for 6,000 population, but I am working with [around] 13,000 people. So, to work in eight subblocks, to visit the ward, visit the house, one motorcycle can be provided either by government or donor. If it is provided, the service will be faster, sounder, and more successfully done by us.* —HA/CHW FGD

Having transportation also would enable CHWs to better mobilize the community and increase awareness about important health topics.

*I have a motorcycle of my own. […] I used a loudspeaker to spread the news of EPI (Expanded Program on Immunization). I drove my motorcycle through the area where EPI will take place and the recorded details about the session was being played through the loudspeaker The recording was in local language and I played the record, drove the bike. After this, there was no such place where the news had not been reached. It was a great advantage. There are some places where rickshaw couldn't go but motorcycle can. The motorcycle is really important here. So I have a request that please deliver this message that motorcycle is really important for HA. I think 100% coverage is achievable if they are provided with a bike.* —HA/CHW FGD

### Provision of Tools and Medical Supplies

CHWs discussed the need for tools and resources that would enable them to work more efficiently, including medicines, logistical support, job aids, and smartphones/tablets. Both CHW cadres want to be able to distribute medicines as they felt that it would not only be an essential and needed service for the community, but it would also strengthen the relationship between CHWs and the community. FWAs currently are not able to distribute medicines outside of family planning methods and vitamin A capsules (when relevant).

*We could supply medicines for common health problems such as gastric, fever, diarrhea, mucus diarrhea, etc. in the field level. People think they can have these medicines for us, but we cannot provide this service.* —HA/CHW FGD

*Providing medicines like iron tablet through FWA would also help to get more respect in the field.*—CHW Supervisor FGD

Given the increasing workloads and the supplies they must carry during their household visits, CHWs discussed that it would be beneficial to digitize aspects of their work. CHWs felt that having a smartphone or a tablet would enable them to work more effectively:

*Our notebooks are getting larger. Our notebooks are at least 3 kg in weight, and it is increasing to size after adding those 19 columns regularly. So, if the extent of our work is increasing so much, why are we not given tabs [tablets]?* —CHW Supervisor FGD

### Training

Both CHW cadres are meant to receive 1 month of “basic” training for their job; however, CHWs reported often not receiving it:

*I've joined in 2016 but haven't got my basic training yet. Even people who have joined back in 2008 didn't get their basic training yet.* —CHW Supervisor FGD^2^

In addition to this, CHWs want to have refresher trainings and new trainings to reinforce and expand upon their knowledge and skills. CHWs reported that trainings on counseling methods, taking blood pressure, different types of medication, vaccinations, and so forth occur infrequently, providing them with little opportunity to update their knowledge. CHWs also desired to receive technical training, which would increase their technical recognition and could improve their salary grades; for example, FWAs wanted technical trainings to become certified community skilled birth attendants. Additionally, CHWs discussed the importance of being trained on how to use digital devices.

*We vaccinate a child from very beginning of life. So, if anything happens to him, the liability is ours. If something happens to any child, whole area would be upside down. We have the liability of child death. In this case, we don't receive much training. Even CHCPs receive 6 months training. But we haven't received any basic training. There are 30 types of medicines here [family welfare centers]. We don't even have any training about them. We are going on with our own experience. No basic training has been done about this. If we receive training, our skill would be more developed.* —HA/CHW FGD

*The problem is, whenever we talk about technical issues, higher officer or line doctor suppress us saying, “What do you want? You didn't join with the educational qualification of a technical [employee].” […] So, we want that when we get appointed, we will be given a one-year technical training for the rank.* —HA/CHW FGD

### Workload

CHWs feel that their workload has become quite burdensome, which has significantly affected their motivation and morale. Given the lack of an adequate CHW to population ratio, the existing CHWs are often overstretched and are responsible for supporting more people than originally intended, which has adversely affected CHWs and raised concerns about their ability to work effectively.

CHWs feel that their workload has become quite burdensome, which has significantly affected their motivation and morale.

*Every ward has 10,000 people. […] For having trouble with manpower, we serve about 20,000 people per unit. Who used to serve three wards in a Union Parishad, now that person has to work for six wards, where it is supposed to be two people per ward. In [Location], we had three person working for health service, but about 50% of manpower is gone [retired] by now. Last appointment was in April 2010. Some people retired or got promoted in the meantime. But no appointment was conducted for these vacant posts.* —HA/CHW FGD

*How am I going to serve them [the community] or counsel them properly? How am I going to reach every mother in my area? I have to reach to them. I have to hurry. Sometimes they request us to stay a bit more but I can't. We are getting hurried from the health complex, we are getting hurried from community clinics, but family planning is not an easy job to do. We are being over-pressured.* —FWA/CHW FGD

### Workplace Environment

Although CHWs primarily work in the community and CCs, there may not always be dedicated space available for them to work. CHWs feel that this needs to be addressed in some capacity because the lack of a dedicated workplace environment adversely affects their performance, as well as their relationships with the community and health care facilities. CHWs desired improvements in 2 types of workplace environments: EPI centers and CCs. For EPI centers, CHWs discussed challenges faced within the community of hosting sessions in people's homes, noting that it places a further burden on community members, which also affects CHWs' ability to provide services if the community member is not willing to share their home. Some CHWs noted that it might be worth paying community members to use their home or renting facilities elsewhere within the community:

*If we could rent their room and do our EPI sessions there, it would be a lot easier. Government doesn't have to make a new facility for us as well. We could keep all our logistics there and prepare ourselves better. Thus, we can develop our relationship between each other.* —CHW Supervisor FGD

At the CCs, CHWs often find there is no space for them to work despite having to provide services from CCs 3 days a week. FWAs noted this problem happened to their cadre frequently. Given that FWAs are expected to be at CCs 3 days out of the week, the lack of a dedicated space for them has become demotivating and insulting. It also further exacerbates tensions between the CHW cadres.

*Now let me talk about the community clinic. It has three rooms: one for CHCP, one for HA, and [the] last one is a balcony. When a FWA brings a mother for check-up in the community clinic, they do not find a private place for the mother. They told us to use the balcony. But how can we use a balcony to check-up [on] the pregnant mother? She deserves some privacy. The HAs use the room for EPI and let the FWAs use the balcony for satellite. There should be a separate room for the FWAs. Our health department has two children, one is his own, the other is his stepson. Family planning sector is [his] step-son.* —CHW Supervisor FGD

### Monetary Factors

#### Salary

As government employees, CHWs receive a monthly salary; however, the amount provided is often not enough. CHWs are discontent with their salary levels. For FWAs, the change in class positions resulted in a decrease of their salary grade. This caused significant upset among FWAs, especially as HAs maintained their higher-grade position.

CHWs receive a monthly salary; however, the amount provided is often not enough, and CHWs are discontent with their salary levels.

*In agriculture department, field workers get second class [13th grade] salary. But we get fourth class [17th grade] salary. Salary discrimination should be eradicated. […] In our union, we have four FWAs though we need nine of them. If one person carry this much pressure, how can success come? FWAs also work in the technical side as they push injection. If their salary could be equal as technical side, it would help to get better outcome from [their] field work.* —CHW Supervisor FGD

For HAs, their salary level remains stagnant despite promotions to higher ranks (e.g., AHI/HI):

*The salary discrimination needs to be taken care of. If an HA gets the same basic of 20,180 Taka, as an AHI or HI gets the [same], then why would he address HI as sir?* —CHW Supervisor FGD

Both of these issues are considerably demotivating and demoralizing for CHWs:

*The salary [the] government gives us, it is not enough. We have to spend our salary to go to the field work and have to come back. It is a long distance. So, I think the first condition is to eradicate the salary discrimination.* —CHW Supervisor FGD

#### Allowances

In addition to their salaries, CHWs also receive allowances to help support their work, particularly for transportation. They receive allowances for accommodation/housing, transportation, child education, and tiffin (i.e., light meal), as well as for 2 yearly festivals and the Bengali New Year. However, the amounts provided are insufficient, with CHWs spending their own money to support their work and their communities. CHWs do receive transportation allowances to attend meetings or take logistics to subdistrict headquarters; however, the allowances are not provided consistently, as CHWs noted having to pay the costs of their transportation to fulfill responsibilities.

*We have to spend a lot of money for transportation. But we don't get any transport allowance for it. In [Location], the FWAs aren't provided transport allowances. We have to spend BDT 150/200 each day for transportation.* —FWA/CHW FGD

*I think we should consider their side financially. They need financial incentives. For example, when a worker of mine is going somewhere, we should pay him the travel allowance that he needs because otherwise he will have to pay it by himself. And it can lessen his motivation to do his best.*—Upazila/District-Level Supervisor IDI

Additionally, CHWs want to see an increase in the tiffin allowances provided. CHWs are often busy with their workload and working on their feet, which limits the time they have for meals; they feel that allowances should be increased to subsidize those costs. CHWs also discussed the need to create several types of allowances for excess workload, risks/emergencies, and tourism. This was especially discussed in Cox's Bazar, where the Rohingya refugees are residing. CHWs in Cox's Bazar have an increased workload, as well as increased risk, given the higher prevalence of diseases within the area. Allowances for risks/emergencies was also a significant need, as CHWs work in environments in which they are often putting themselves at potential risk for exposure to illness and injury:

*We don't even get any risk allowance. Let's say I am injecting medicine to an AIDS patient; it can spread to me via syringe. I face a continuous risk of getting infected. I need my risk allowance* —HA/CHW FGD

Tourism allowance was specifically seen in discussions in Cox's Bazar; CHWs cited the high cost of living due to Cox's Bazar also being a tourist destination.

## DISCUSSION

Our study identifies several challenges that are unique to and have direct implications for the health care sector in Bangladesh. First, tension between HAs and FWAs, owing to the differences in their government technical grade and salary scales despite comparable responsibilities, undermines the motivation of the CHWs. In practice, minimal differences exist in the responsibilities and the workload of FWAs and HAs, yet HAs are placed at a higher technical rank and salary bracket. Changes to technical ranks and wages for the health care sector need to take a sector-wide lens so that wages for all health care workers can be systematically calibrated.

Second, the study highlights the importance of the workplace environment in fostering motivation and the ability to execute responsibilities effectively. The scope of activities undertaken by CHW cadres have evolved over the last decade, without the necessary attention to how they will be executed. FWAs are required to spend a few days a week at the CCs, however, they lack dedicated physical spaces where they can conduct clinical assessments, many of which require privacy. HAs often conduct their EPI sessions in community members' homes and face challenges when those homes are not available to them. CHWs noted that this has often caused strain within the community-CHW relationship. As such, it may be important to consider possible alternatives such as renting a community member's home or another existing community-owned location. Discrepancies regarding dedicated workspace also highlighted tensions between cadres, as some FWAs reported the lack of a dedicated workspace and disregard for their cadre in the CCs due to the differences in government rank and salary scales.

Third, CHWs continue to be seen as a quick-fix solution to emergent health challenges, including chronic disease, a refugee health crisis, and a global pandemic. CHWs who serve the community in Cox's Bazar highlighted the unique challenges and risks they face in serving the large Rohingya refugee settlement. This is also being highlighted globally in the context of COVID-19 as CHWs are serving to identify positive cases and conduct contact tracing. As revisions to CHW and health care worker compensation structures are considered, provisions need to be in place to account for adequate training and risk allowance. This is of particular importance for CHW cadres because they are typically deployed to respond to emergent situations without adequate investment in preparing or supporting them. Any changes to CHWs' scope of work need to take into account the burden of work they are required to undertake versus the time allocated for them to do it.^21^ As community change agents, it is expected that CHWs will be required to respond to the needs and priorities of the community; however, the ever-expanding tasks need to be routinely assessed against what is feasible.

The scope of activities undertaken by CHW cadres have evolved over the last decade, without necessary attention to how they will be executed.

The study also identifies other programmatic challenges faced by the CHW cadres in Bangladesh—inadequate recognition, means of identification and transportation, access to working tools, and training opportunities—that have already been extensively documented in the literature related to CHW programs globally.^7^^,^^22^^–^^25^ Bangladesh is unique in having multiple cadres of CHWs that are scaled nationally and institutionalized within the Ministry of Health with a formal technical rank, salary, and pension. Global advocacy efforts to institutionalize CHWs are often focused on the need for formal recognition as government employees, as well as salaries and other benefits accrued because of government employment.^8^^,^^26^^–^^29^ However, in the case of Bangladesh, we see that the mature institutionalized CHW cadres face many of the same challenges faced by fledgling cadres in other countries. These systemic challenges have downstream effects on how effective the CHW programs are, as well as on the health of the community.

The case of Bangladesh shows that mature institutionalized CHW cadres face many of the same challenges faced by fledgling cadres elsewhere.

We argue that institutionalizing CHWs, while valuable, is insufficient for improving the quality of such programs without adequate and sustained investments in the necessary ecosystem that supports such programs. As identified in the CHW logic model, mobilizing inputs at the policy, funding, and organizational level toward a stronger health system is indispensable for high-performing CHW programs.^30^ Fundamentally, to be effective, CHWs need adequate training, opportunities to receive in-service training to maintain and upgrade their knowledge, consistent access to necessary logistics and supplies that will facilitate their job, supervision, appropriate means of transportation, and a compensation structure that is balanced with their workload. Often, donor investments for CHW programs support training only in a priority health area such as family planning or HIV, without any consideration of or investments in the supporting ecosystem that is critical to the effectiveness of these programs. Our study highlights that even in the case of mature, institutionalized CHW cadres, there are no shortcuts to achieving community-level impact. Governments must continue to plan for sustained investments not only in the direct salaries and benefits of the health care workers but also in ensuring that systems are in place for adaptive management of the CHW cadres based on changing community needs, assessing performance, and aligning support with the expected output. Greater coordination is needed at a global level to pool and align donor investments to facilitate appropriate support for the ecosystem that facilitates the success of CHW programs.

The study identifies several factors that impede the effectiveness of the CHW programs in Bangladesh. The results of our study are consistent with findings from the literature showing the importance of considering both nonmonetary and monetary incentives to support CHWs.^8^^,^^26^^,^^28^^,^^29^^,^^31^^–^^35^ The study provides perspectives from CHWs from 4 geographically distinct districts in Bangladesh, including a district with a predominantly refugee population, furthering understanding of factors that ultimately affect CHW job satisfaction and community value for their work. Despite being scaled and institutionalized cadres, the challenges identified are like those in countries with fledgling CHW programs. This study highlights that institutionalization of CHWs without adequate and sustained support for continued training, compensation, supervision, access to working tools, and recognition is insufficient to drive change. Identifying pragmatic strategies that can be supported through existing government budgets to address these factors is vital to sustaining the community health workforce in Bangladesh.

## Notes

**Peer Reviewed**

## References

[1] World Health Organization (WHO). 2018 Health SDG Profile: Bangladesh. WHO; 2018. Accessed August 27, 2021. https://apps.who.int/iris/bitstream/handle/10665/276833/sdg-profile-Bangladesh-eng.pdf

[2] Afsana K, Alam MA, Chen N, et al. Community Health Workers in Bangladesh. Exemplars in Global Health; 2020. Accessed August 27, 2021. https://www.exemplars.health/topics/community-health-workers/bangladesh

[3] Government of Bangladesh. Ministry of Health and Family Welfare (MOHFW). Bangladesh National Strategy for Community Health Workers. MOHFW; 2019. Accessed August 27, 2021. http://www.communityclinic.gov.bd/admin/content_uploads/CHW%20strategy.pdf

[4] Ahmed SM Evans TG Standing H Mahmud S. Harnessing pluralism for better health in Bangladesh. Lancet. 2013;382(9906):1746–1755. 10.1016/S0140-6736(13)62147-9. 24268003

[5] World Health Organization (WHO). WHO Guideline on Health Policy and System Support to Optimize Community Health Worker Programmes. WHO; 2018. Accessed August 27, 2021. http://apps.who.int/iris/bitstream/handle/10665/275474/9789241550369-eng.pdf 30431747

[6] Rahman SM Ali NA Jennings L. Factors affecting recruitment and retention of community health workers in a newborn care intervention in Bangladesh. Hum Resour Health. 2010;8(1):12. 10.1186/1478-4491-8-12. 20438642 PMC2875202

[7] Alam K Tasneem S Oliveras E. Retention of female volunteer community health workers in Dhaka urban slums: a case-control study. Health Policy Plan. 2012;27(6):477–486. 10.1093/heapol/czr059. 21900361

[8] Ormel H Kok M Kane S. Salaried and voluntary community health workers: exploring how incentives and expectation gaps influence motivation. Hum Resour Health. 2019;17(1):59. 10.1186/s12960-019-0387-z. 31324192 PMC6642499

[9] El-Saharty S Sparkes SP Barroy H Ahsan KZ Ahmed SM. The Path to Universal Health Coverage in Bangladesh: Bridging the Gap of Human Resources for Health. The World Bank; 2015. 10.1596/978-1-4648-0536-3

[10] World Health Organization (WHO) Regional Office for South-East Asia. Expanded Programme On Immunization (EPI) factsheet 2019: Bangladesh. WHO; 2019. Accessed August 27, 2021. https://apps.who.int/iris/handle/10665/329976

[11] Riaz BK Ali L Ahmad SA Islam MZ Ahmed KR Hossain S. Community clinics in Bangladesh: a unique example of public-private partnership. Heliyon. 2020;6(5):e03950. 10.1016/j.heliyon.2020.e03950. 32420500 PMC7218291

[12] World Health Organization (WHO) Regional Office for the Western Pacific. Bangladesh Health System Review. Health Systems in Transition vol. 5, no. 3. WHO Regional Office for the Western Pacific; 2015. Accessed August 27, 2021. https://iris.wpro.who.int/handle/10665.1/11357

[13] Alam K Tasneem S Oliveras E. Performance of female volunteer community health workers in Dhaka urban slums. Soc Sci Med. 2012;75(3):511–515. 10.1016/j.socscimed.2012.03.039. 22595068

[14] Ballard M Montgomery P. Systematic review of interventions for improving the performance of community health workers in low-income and middle-income countries. BMJ Open. 2017;7(10):e014216. 10.1136/bmjopen-2016-014216. 29074507 PMC5665298

[15] World Health Organization, UNICEF. Declaration of Astana: Global Conference on Primary Healthcare. WHO; 2018. Accessed August 27, 2021. https://www.who.int/docs/default-source/primary-health/declaration/gcphc-declaration.pdf

[16] Information Technology Outreach Services (ITOS). Bangladesh—Subnational Administrative Boundaries Dataset. ITOS; 2015.

[17] Milton A Rahman M Hussain S. Trapped in statelessness: Rohingya refugees in Bangladesh. Int J Environ Res Public Health. 2017;14(8):942. 10.3390/ijerph14080942. 28825673 PMC5580644

[18] Rohingya emergency. United Nations High Commissioner for Refugees USA. Accessed August 27, 2021. https://www.unhcr.org/en-us/rohingya-emergency.html

[19] QSR International Pty Ltd. NVivo Qualitative Data Analysis Software. QSR International Pty Ltd; 2018.

[20] Boyatzis R. Transforming Qualitative Information: Thematic Analysis and Code Development. Sage; 1998.

[21] Morrow M Sarriot E Nelson AR. Applying the community health worker coverage and capacity tool for time-use modeling for program planning in Rwanda and Zanzibar. Glob Health Sci Pract. 2021;9(Suppl 1):S65–S78. 10.9745/GHSP-D-20-00324. 33727321 PMC7971371

[22] Takasugi T Lee ACK. Why do community health workers volunteer? A qualitative study in Kenya. Public Health. 2012;126(10):839–845. 10.1016/j.puhe.2012.06.005. 23036777

[23] Condo J Mugeni C Naughton B. Rwanda's evolving community health worker system: a qualitative assessment of client and provider perspectives. Hum Resour Health. 2014;12(1):71. 10.1186/1478-4491-12-71. 25495237 PMC4320528

[24] Greenspan JA McMahon SA Chebet JJ Mpunga M Urassa DP Winch PJ. Sources of community health worker motivation: a qualitative study in Morogoro Region, Tanzania. Hum Resour Health. 2013;11(1):52. 10.1186/1478-4491-11-52. 24112292 PMC3852396

[25] George MS Pant S Devasenapathy N Ghosh-Jerath S Zodpey SP. Motivating and demotivating factors for community health workers: a qualitative study in urban slums of Delhi, India. WHO South East Asia J Public Health. 2017;6(1):82–89. 10.4103/2224-3151.206170. 28597864

[26] Brunie A Wamala-Mucheri P Otterness C. Keeping community health workers in Uganda motivated: key challenges, facilitators, and preferred program inputs. Glob Health Sci Pract. 2014;2(1):103–116. 10.9745/GHSP-D-13-00140. 25276566 PMC4168609

[27] Kok MC Kane SS Tulloch O. How does context influence performance of community health workers in low- and middle-income countries? Evidence from the literature. Health Res Policy Syst. 2015;13(1):13. 10.1186/s12961-015-0001-3. 25890229 PMC4358881

[28] Kasteng F Settumba S Källander K Vassall A; inSCALE Study Group. Valuing the work of unpaid community health workers and exploring the incentives to volunteering in rural Africa. Health Policy Plan. 2016;31(2):205–216. 10.1093/heapol/czv042. 26001813 PMC4748129

[29] Geldsetzer P De Neve JW Boudreaux C Bärnighausen T Bossert TJ. Improving the performance of community health workers in Swaziland: findings from a qualitative study. Hum Resour Health. 2017;15(1):68. 10.1186/s12960-017-0236-x. 28923076 PMC5604406

[30] Naimoli JF Frymus DE Wuliji T Franco LM Newsome MH. A community health worker “logic model”: towards a theory of enhanced performance in low- and middle-income countries. Hum Resour Health. 2014;12(1):56. 10.1186/1478-4491-12-56. 25278012 PMC4194417

[31] Kok M Abdella D Mwangi R. Getting more than “claps”: incentive preferences of voluntary community-based mobilizers in Tanzania. Hum Resour Health. 2019;17(1):101. 10.1186/s12960-019-0438-5. 31847909 PMC6918602

[32] Scott K Beckham SW Gross M. What do we know about community-based health worker programs? A systematic review of existing reviews on community health workers. Hum Resour Health. 2018;16(1):39. 10.1186/s12960-018-0304-x. 30115074 PMC6097220

[33] Kok MC Dieleman M Taegtmeyer M. Which intervention design factors influence performance of community health workers in low- and middle-income countries? A systematic review. Health Policy Plan. 2015;30(9):1207–1227. 10.1093/heapol/czu126. 25500559 PMC4597042

[34] Mays DC O'Neil EJ Jr Mworozi EA. Supporting and retaining Village Health Teams: an assessment of a community health worker program in two Ugandan districts. Int J Equity Health. 2017;16(1):129. 10.1186/s12939-017-0619-6. 28728553 PMC5520299

[35] Saran I Winn L Kipkoech Kirui J Menya D Prudhomme O'Meara W. The relative importance of material and non-material incentives for community health workers: evidence from a discrete choice experiment in Western Kenya. Soc Sci Med. 2020;246:112726. 10.1016/j.socscimed.2019.112726. 31869666

